# Assessment of Tumor Angiogenesis by Expression of CD 105 in Ameloblastoma, Odontogenic Keratocyst and Central Giant Cell Lesion

**DOI:** 10.31557/apjcp.2020.21.11.3373

**Published:** 2020-11

**Authors:** Khurshid Ali, Sultan Zeb Khan, Nuzhat Sultana, Osama Alghamdi, Samrina Mohammad, Sameer A Mokeem, Saqib Ali, Tariq Abduljabbar, Fahim Vohra

**Affiliations:** 1 *Department of Oral Pathology, Khyber College of Dentistry, Peshawar, Pakistan. *; 2 *Department of Pathology, Northwest School of Medicine, Peshawar, Pakistan. *; 3 *Department of Oral and Maxillofacial Surgery, College of Dentistry King Saud University. Riyadh, Saudi Arabia. *; 4 *Department of Periodontics and Community Dentistry, College of Dentistry, King Saud University. Riyadh, Saudi Arabia.*; 5 *Department of Biomedical Dental Sciences, College of Dentistry, Imam Abdulrahman Bin Faisal University, Dammam, Saudi Arabia. *; 6 *Department of Prosthetic Dental Science, College of Dentistry, King Saud University, Research Chair for Biological Research in Dental Health, Riyadh, Saudi Arabia. *

**Keywords:** Blood vessels, endoglin, giant cell granuloma, pathologic angiogenesis, odontogenic tumors

## Abstract

**Background:**

Angiogenesis is critical for tumor growth and reflects the aggressive behavior of invasive odontogenic lesions [like amelogenesis (AM) (AM), Odontogenic Keratocyst (OKC) and Central giant cell lesion (CGCL)]. Mean vascular density (MVD) shows the angiogenic potential and CD105 is an ideal endothelial biomarker due to its specificity to new blood vessels for MVD detection. The aim of the study was to compare the MVD (angiogenic potential) among AM, OKC and CGCL in comparison to Pyogenic Granuloma (PG) using CD105 biomarker.

**Methods:**

Sixty-four primary cases of odontogenic invasive tumors (AM, OKC and CGCL) and PG, diagnosed clinically and histologically were included in the study, with 16 samples in each group. Tissue samples of peripheral AM, Peripheral GCL of jaws, malignant AM, and specimen with insufficient tissue were excluded. Tissue sections were embedded, processed and stained using Hematoxylin and Eosin (H and E). Immunohistochemistry was performed using antibodies against CD105, with positive brown cytoplasmic staining in the endothelial cells of neo-vasculature. Distinct countable, positively stained endothelial cell or clusters were evaluated under light microscope for identification of MVD. ANOVA and t-test were applied for statistical analysis of data.

**Results:**

Highest MVD was displayed in CGCL (32.99±0.77) and the minimum was observed in OKC (7.21± 0.75) respectively. CGCL showed significantly higher MVD to AM, OKC and PG lesions (p<0.05). AM (8.07± 0.36) and Odontogenic Keratocyst (7.21± 0.75) showed comparable MVD, which was lower than PG (14.7± 0.96) and CGCL vascular density (p < 0.01) respectively.

**Conclusion:**

CGCL was most aggressive, with highest MVD among the investigated odontogenic lesions (OKC, AM and PG). The proliferative aggressive behavior of Odontogenic Keratocyst is comparable to AM due to comparable mean vascular density.

## Introduction

The benign odontogenic tumors and cysts of the jaws exhibit locally invasive behavior, and multilocular lesions like Ameloblastoma (AM), Odontogenic Keratocyst (OKC) and central giant cell lesions (CGCL) are comparatively aggressive than unilocular lesion (Gaonkar et al., 2016, Sabino-Bezerra et al., 2013,). Among the odontogenic lesions, CGCL commonly manifest as loculated radiolucency in the anterior mandible of young females (Whitaker and Bouquot, 1994). They are characterized with the presence of multinucleated giant cell granuloma, mononuclear stromal cells with intra-osseous origin (Amaral et al., 2010). Biological behavior of CGCL ranges form slow growth to aggressive destructive expansion, manifesting as cortical bone perforation and root resorption (Whitaker and Waldron, 1993). 

AM is the most commonly occurring epithelial odontogenic tumor, which is locally invasive with continuous growth and high level of recurrence (Vohra et al., 2009). Originating from the remains of dental lamina, cystic lining and basal cell layer, they can manifest in unicystic, multicystic, peripheral and malignant forms; and its histologic variants include plexiform, follicular, granular, desmoplastic, acanthomatous, basal cell and clear cell types (Sisto and Olsen, 2012). Multilocular AM are one of the most prevalent (65%) variants, however exhibit low malignant transformation (5%) and are mostly treated with marginal resection (Sharifi-Sistani et al., 2011, Gomes et al., 2010, Van Dam et al., 2010). Another odontogenic lesion in the differential diagnosis of AM with high recurrence rate is Odontogenic Keratocyst (Keratocystic odontogenic tumor-OKC). Originating from the basal epithelial cells, it is characterized by proliferation in epithelium, para-keratinization in the surface layer and high mitotic rate associated with genetic abnormalities (mutation in p53, tumor suppressor gene) (Alaeddini et al., 2009). OKC appears to be a cystic lesion, however it resembles AM in its invasive, aggressive behavior (Alaeddini et al., 2009). At the center of attention are the microscopic epithelial and stromal features in investigating the behavior of these odontogenic but locally invasive lesions (OKC, AM, CGCL).

Odontogenic epithelial cells are devoid of a vascular system, and connective tissue stroma supports epithelial changes and expansion through angiogenesis. Absence of nutrients and oxygen from lack of neo-vascularization results in epithelial cell apoptosis in tumors. The presence of myofibroblasts and blood vessels in connective tissue stroma is pivotal to the neoplastic growth; and angiogenesis is fundamental to it. Angiogenesis is a process of new blood vessel formation from pre-existing vessels, involving a sequence of events. The spectrum of processes include proliferation and endothelial cell migration, proteolytic breakdown of extracellular matrix, capillary formation, loop and lumen development and anastomosis of micro vessels (Hande et al., 2011). The process is regulated with the help of growth factors and inhibitory biomarkers, (Margaritescu et al., 2010). An ideal marker for angiogenesis should detect quality and quantity of newborn vessels, and CD105 is expressed differentially in angiogenesis in contrast to normal endothelial cells (CD31 and CD34) (Hande et al., 2011, Gadbail et al., 2011b). 

As angiogenesis reflects the growth potential of odontogenic tumors, assessment of mean vascular density helps to predict the behavior of the lesions as investigated previously (Kumar et al., 2016, Gadbail et al., 2011b, Jamshidi et al., 2014). Kumar et al., reported that AM and Odontogenic Keratocyst (OKC) showed high mean vascular density (MVD) of 7.98±2.70 and 6.25 ±2.88 respectively, while a lower mean value of 3.75±1.42 was exhibited by Dentigerous cyst (DC) (Kumar et al., 2016). They concluded that AM and OKC did not show a statistically significant difference (p > 0.05). Gadbail et., (2011b) reported that OKC had a significantly higher MVD than DC and normal mucosa (P<0.05). They concluded that CD105 was strongly expressed in microvessels of odontogenic keratocyst as compared to those of normal oral mucosa and dentigerous cyst. However, Jamshidi et al reported that CD105 and CD34 markers showed significant differences statistically in MVD in AM and the OKC groups (p= 0.005, and p= 0.000) respectively (Jamshidi et al., 2014). AM showed higher MVD as compared to odontogenic Keratocyst (Jamshidi et al., 2014). There appears to be a controversy in the MVD of Odontogenic tumors in published literature. It is hypothesized that MVD among odontogenic tumors including AM, OKC, CGCL, and in comparison to pyogenic granuloma will be significantly different. Therefore the purpose of this clinical and experimental study was to compare the mean vascular density using CD105 biomarker, among the samples of AM, Odontogenic keratocyst and Central Giant Cell Lesions in comparison to Pyogenic Granuloma to ascertain common behaviors of these tumors in population. 

## Materials and Methods

*Ethical approval and study setting*

The study was approved by the Ethics and Research Committee of Gandhara University and was performed following the standards and protocols of Revised Helsinki declaration (2014). It was a cross-sectional comparative (analytical) study conducted at the Dental school, medical and histopathology research laboratory from December 2018 to May 2019. 

*Study population and criteria*

A sample size of 64 human subjects was considered appropriate, which was calculated from means and standard deviations (6.25±2.88 SD in group 1 and 3.75±1.42 SD in group 2) of a previous study by using openepi calculator (Bosari et al., 1992). The required sample size (convenience sampling) for test and control groups was 16 at 80% power. Pre-diagnosed, formalin-fixed, paraffin-embedded tissue samples of unicystic and solid AM, parakeratinized OKC, and CGCL of jaws were included in the study. Tissue samples of peripheral AM, Peripheral CGCL of jaws, malignant AM, and specimen with insufficient tissue were excluded. As a result, there were four different groups of jaw lesions including AM (Group AM), Odontogenic Keratocyst (Group OKC), Central Giant Cell Lesions of jaws (Group CGCL), and Pyogenic Granuloma (Group PG). A total of 64 formalin-fixed, paraffin-embedded tissue samples of AM, OKC, CGCL of jaws, and PG were collected and mounted on slides by consecutive, non-probability sampling technique. The slides were labeled, allotted specific numbers for identification, and were stained by H and E, and CD105 antibody at Histopathology Research Laboratory. 

Two slides were prepared for each tissue sample. To compare one slide stained with H and E, and others reacted with CD105 antibody (Dako) using immunohistochemistry.

*Hematoxylin and Eosin staining*

Slides with tissue sections were placed in a hot air oven at 60oC for 60 min (minutes) and were deparaffinized with 2 changes of xylene, 5 min each. For dehydration, the slides were immersed in 100%, 90%, 80% and 70% ethanol for 2 min each respectively. Following which the slides were immersed in tap water for 3 min and given 3 changes. Following with a dip in Harris Haematoxylin for 10 min and washed well in running tap water for 5 min. Preparation continued with immersion in 1% acid alcohol for 2 secs, washed in water for 5 min until the sections became blue, then immersed in 1% ammonia water for 5 secs, washed in tap water for 5 min. Specimens were immersed in 1% eosin for 2 min, followed with 70% and 100% ethanol for 2 min each with water washing (2 min) in the end. Lastly slides were immersed in xylene for 2 min and mounted with Dibutylphthalate Polystyrene Xylene (DPX).

*Immunohistochemistry*

In this study CD 105 antibody was applied to the tissue sections employing immunohistochemistry. One section from each block, of 3-4 µm thickness was taken on Super-frosted slides after coating them with Poly-L-lysine. These sections were reacted with the CD105 antibody (Dako) using the avidin-biotin-peroxidase complex method. In this method, the sections were dried at 60°C for 60 min in a hot air oven then dewaxed by the sequence of immersion in xylene twice for 5 min, 100% and 95% ethanol for 3 min twice each, and washing with distilled water for 2 min twice.

To retrieve antigen, application of proteinase k at 37°C temperature for 30 min was performed. Washing with water (10 min) was performed followed by wash with PBS for 2 mints each with 3 changes. 1 to 2 drops of hydrogen peroxidase block were applied on the slide, incubated (10 min) and PBS washing. This followed placement in buffer bath for 2-3 min. Primary antibodies were diluted according to the suggested dilutions as 1:10 by the manufacturer (Dako). 2 drops of primary antibody were placed on the slides and incubated for 1 hour. This followed application of linker solution for 30 min and placement again in buffer bath for 2-3 min. Processing also included application of biotinylated secondary antibody reagent (10 min) and streptavidin peroxidase reagent (10 min) followed by PBS wash and buffer bath. Finally, substrate chromogenic solution was applied (10 min) and later washed. Prior to assessment, slides were dehydrated with immersion in 70%, 90% and 100% ethanol for 5 min each respectively, followed by Xylene immersion (5 min) and mounting using DPX. 

*Microscopic interpretation*

Stained sections were interpreted microscopically under the light microscope (Olympus CX 31). The presence and frequency of micro vessels were identified as vascular endothelial cells, which were stained by CD105, easily recognized by brown cytoplasmic staining. The criteria laid down by Weidner (Weidner et al., 1993), was adopted and only strong positively stained endothelial cell or cell cluster, which was easily separated from adjacent micro vessel and other connective tissue elements were considered as a distinct countable micro vessel. Ten regions under x100 magnification with the high number of vessels were selected. In each of 10 fields, micro vessels were counted at x400 magnification which is approximately 0.18 mm2 field size. Two operators simultaneously evaluated all the slides by double head microscope (KA and SA). They both had to agree on each micro vessel before it was included in the count. The mean vascular density was calculated as a mean number of vessels per high power field and then documented in the prescribed data collection proforma. 

*Statistical analysis*

Data was analyzed using Statistical program for social sciences (SPSS, version 20). MVD of each lesion was compared with PG (control) using an independent T-test. ANOVA was applied for to compared outcomes among the groups and post hoc multiple comparisons test was employed to see comparison between individual groups, with p < 0.05 as statistically significant.

## Results

The mean vascular density data was normally distributed, as assessed with Kolmogorov-Smirnov Test. The maximum MVD was displayed in central giant cell lesion (32.99±0.77) and the minimum was observed in OKC (7.21± 0.75) respectively. Mean vascular density among AM samples was 8.07± 0.36, however among the control specimens of pyogenic granuloma MVD was 14.7± 0.96 (Table 1). 

Comparison of MVD among the tested lesion groups (AM, OKC, CGCL and PG) showed a significant difference (p< 0.05), as shown in Table 1. Pairwise comparison of control specimens (Pyogenic granuloma) showed significantly higher MVD as compared to AM (p < 0.01) and OKC (p <0.01) (Table 2). However PG specimens showed lower MVD as compared to Giant cell lesions (p <0.01). AM and OKC showed comparable MVD, which was lower than Giant cell lesion MVD (p < 0.01). 

**Figure 1:**
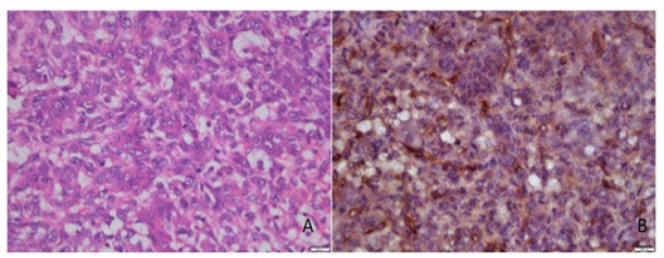
Photomicrograph of H & E Staining (A) and Immunohistochemical staining (B) with CD105, of micro vessels around giant cells in a Central giant cell lesion section

**Figure 2:**
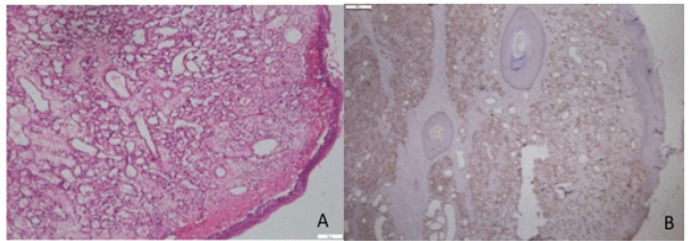
Photomicrograph of H & E Staining (A) and Immunohistochemical staining (B) with CD105, of micro vessels in a Pyogenic granuloma section

**Figure 3:**
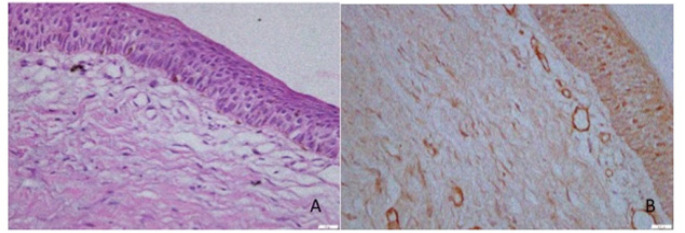
Photomicrograph of H & E Staining (A) and Immunohistochemical staining (B) with CD105, of micro vessels in connective tissue wall beneath epithelium in a Odontogenic Keratocyst section

**Figure 4:**
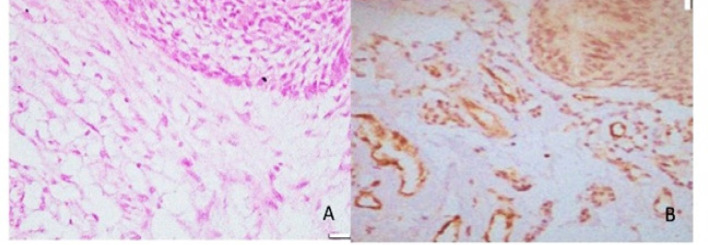
Photomicrograph of H & E Staining (A) and Immunohistochemical staining (B) with CD105, of micro vessels adjacent to epithelial islands in an AM section

Figures 1 to 4 present the histological findings of the H and E and CD 105 staining indicating the different mean vascular densities among the different lesions. The most dense appearance of vessels from brown staining of endothelial cells was presented by Giant cell lesion (Figure 1). The blood vessels were large in number and in smaller sizes adjacent to giant cells compared to other lesions (Figure 1- CGCL). The specimens of pyogenic granuloma also showed relatively high density of CD 105 stained micro vessels through endothelial cell staining, showing widely dispersed medium size micro vessels (Figure 2). In OKC specimens, the field for CD105 strongly positive vessels was selected below the epithelial lining showing fewer and larger blood vessels in the connective tissue wall (Figure 3). AMs however showed nests, islands, and the cords of the epithelial tissue and beneath them relatively moderate number of micro vessels (Figure 4). Micro vessels in AM were normal sized, well-distributed and also revealed anastomosis, especially around tumor islands.

**Table 1:** Means and Standard Deviations of Mean Vascular Density (MVD) among Compared Lesions

Lesion Type	Mean	SD	95% CI	Range	P Value*
AM	8.07 ^a^	0.36	(7.87, 8.26)	7.3- 8.8	
OKC	7.21 ^a^	0.75	(6.80, 7.60)	5.5-8.2	< 0.05
GCL	32.99 ^b^	0.77	(32.60, 33.38)	31.6 -34.4	
PG (control)	14.7 ^c^	0.96	(14.24, 15.15)	13.3-17.2	

AM, Ameloblastoma; OKC, Odontogenic Keratocyst; GCL, Giant cell lesion; PG, Pyogenic granuloma

**Table 2:** Statistical Comparison of the Mean Vascular Density (MVD) of AM, OKC and GCL with Pyogenic Granuloma (Control)

Lesions compared	Paired Differences				P value
	Mean	SD	95% Confidence Interval of the Difference		
			Lower	Upper	
PG vs AM	-6.55000	0.91652	-7.03838	-6.06162	P <0.01
PG vs OKC	-7.41250	1.21922	-8.06218	-6.76282	P<0.01
PG vs GCL	-18.30625	1.28035	-18.98850	-17.62400	P<0.01

AM, Ameloblastoma; OKC, Odontogenic Keratocyst; GCL, Giant cell lesion; PG, Pyogenic granuloma

## Discussion

The present study aimed to investigate the mean vascular density among the invasive jaw lesions including AM, OKC and CGCL using immunohistochemistry (CD105- Endoglin) as a marker of angiogenesis. It was hypothesized that MVD among AM, OKC, CGCL, and in comparison to pyogenic granuloma will be significantly different. Giant cell lesions showed the maximum MVD, which was greater than AM and OKC, indicating the invasive expansive behavior of CGCL in jaws. Therefore the study hypothesis was accepted.

CD105 (Endoglin) expression is a prominent feature of newly formed blood vessels. It is commonly identified through brown staining of the endothelial cells cytoplasm and is employed for diagnosis, therapeutic response and prognosis of lesions (Nikiteas et al., 2007, Kumar et al., 2015, Gadbail et al., 2011b). The marker binds several factors of the Transforming Growth Factor (TGF-β) superfamily, which is a pleiotropic cytokine, regulating different functions of the cell including proliferation, differentiation, and migration (Nikiteas et al., 2007). Tumor growth requires the formation of blood vessels for oxygen and other nutrients; therefore CD105 is a critical marker for the quality and quantity detection of newborn vessels specific to tumors (Nagatsuka et al., 2005, Jamshidi et al., 2014, Bosari et al., 1992). In contrast to pan-endothelial markers like von Willebrand, factor VIII, CD31, and CD34, the expression of CD105 is insignificant in previously formed vessels, endothelial cells of lymphatic vessels and endothelium of normal tissues (Fonsatti and Maio, 2004). In the present study, MVD assessment was based on Widener’s criteria1, defining distinct micro vessels as endothelial cells with a vascular lumen or cell clusters separated from adjacent micro vessels. As adopted from previous studies (Gadbail et al., 2011a, Kumar et al., 2015), Single-cell sprouts with no lumen were included in micro vessel identification process. Moreover, pyogenic granuloma lesion was considered as control to compare with invasive lesions (AM, OKC and GCL). However previous studies have used PG in addition to normal oral mucosa as control specimens (Kumar et al., 2015, Gadbail et al., 2011b). 

In the present study, CGCL showed higher vascular density than PG (control). Central giant cell lesions occur twice as often in the mandible than the maxilla, and commonly in the anterior compared to posterior region (Bocchialini., 2020, Khanna et al., 2018, Whitaker and Waldron, 1993). In addition, giant cell lesions are comprised of fibrocellular stroma with mononuclear cells, characterized by presence of ovoid and spindle shaped cells indicative of the activity and proliferation in the tumor (Chang et al., 2016, Chaparro-Avendao et al., 2005). It is suggested that proliferative activity and vascularity have a vital role in tumor growth and invasiveness (Kumar et al., 2016). The rate of angiogenesis in the tumor is associated with the clinical outcomes, which suggest that properties of angiogenesis correlate with tumor behavior, influencing the architecture and pattern of tumor growth. Mean vascular density is commonly used as a critical prognostic marker of neoplasms; therefore our findings indicate that central giant cell granuloma is more aggressive and invasive than AM, OKC and PG (Maeda et al., 1995). In the present study, mean vascular density for Central Giant Cell Lesion was 32.99±0.77, this is in line with the findings of Hallikeri et al. (MVD-34.7±7.14), who used CD34 as a marker of angiogenesis (Hallikeri et al., 2015). Furthermore, macrophages are associated with the angiogenesis in tumor stroma, thereby stimulating and modulating angiogenic process. This is supported by the high presence of macrophages in giant cell lesions with comparative ratios to blood vessels (Hallikeri et al., 2015).

Interestingly, MVD of AM was higher than OKC, however statistically similar; and lower compared to PG in the present study. Specimens of AM reported a MVD of 8.07±0.36, while Odontogenic Keratocyst exhibited vascular density of 7.21±0.75 in the present study. In a study by Kumar et al., (2015) MVD of AM and OKC was 7.98±2.70 and 6.25±2.88 respectively. These findings are similar with the observations in the present investigation. In addition, a similar study compared angiogenesis among OKC, Dentigerous cyst and AM, concluding that MVD was higher in AM compared to Keratocystic odontogenic follicular cyst (Alaeddini et al., 2009). Although the micro vessels were comparatively more in AM, they were anastomosed and smaller than the fewer OKC vessels, which were larger. Odontogenic tumors including AM are locally invasive and matrix metalloproteinase (MMP) expression in the connective tissue stroma is pivotal to it. MMP expression is essential for angiogenesis and its expression is associated with higher angiogenesis in lesions with clinically aggressive behavior (Ali, 2008, Jiang et al., 2008). The findings of MVD in the present study are reflective of the locally invasive and aggressive behavior of OKC, comparable to AM. In addition to density of vessels, the zone of vascular density in the tumor tissue is considered critical in odontogenic tumors. In a study by Seifi et al., (2011) immunohistochemistry showed increase in intra-mural vascular density and angiogenesis, however angiogenesis rate was low in the peritumoral area of AM. However, due to the invasive and recurrent nature of neoplastic lesions, high peritumoral vascular density has been reported in previous studies (Martano et al., 2015). Therefore it is critical to understand that epithelium in odontogenic tumors induces angiogenesis in connective tissue and build up of blood vessels around the odontogenic epithelium for nutrients and oxygen facilitates tumor growth.

Based on angiogenesis, giant cell lesions were more aggressive compared to pyogenic granuloma, AM and OKC due to higher MVD in the present study. In addition, proliferative behavior of OKC was comparable to AM. Therefore, from a clinical perspective, early diagnosis and detection is key to prevent aggressive lesions. In addition, disciplined strict follow-up of suspected lesions with biopsy (identification of angiogenic potential) is critical in early intervention and management of these aggressive lesions. However it is pertinent to mention that other characteristics including, lesion type and classification, stromal cell type, macrophage activity and patient characteristics (age, gender, location) influence the behavior and prognosis of odontogenic lesions. Moreover, multiple biomarkers have been employed in the assessment of angiogenesis [CD 105, CD 34 and Vascular endothelial growth factor (VEGF)] in odontogenic tumors, and various laboratory techniques have been used (Seifi et al., 2011, El‚Labban and Aghabeigi, 1990, Rubini et al., 2011). As methodological heterogeneity can influence the MVD outcomes, therefore multicenter, RCT studies evaluating multiple angiogenic markers for different Odontogenic tumor types using standardized technqiues to assess their neoplastic behavior and prognostic performance are recommended. 

In conclusion, in summary, locally invasive growth pattern and aggressive nature of central giant cell granuloma and AM are positively correlated to CD105 (a marker for MVD). CGCL was most aggressive, with highest MVD among the investigated odontogenic lesions (OKC, AM and PG). The proliferative aggressive behavior of Odontogenic Keratocyst is comparable to AM due to comparable mean vascular density.
